# Impact of Sleep-Disordered Breathing on Heart Rate Turbulence in Heart Failure Patients

**DOI:** 10.1371/journal.pone.0101307

**Published:** 2014-06-26

**Authors:** Akiomi Yoshihisa, Satoshi Suzuki, Mai Takiguchi, Takeshi Shimizu, Satoshi Abe, Takamasa Sato, Takayoshi Yamaki, Koichi Sugimoto, Hiroyuki Kunii, Kazuhiko Nakazato, Hitoshi Suzuki, Shu-ichi Saitoh, Yasuchika Takeishi

**Affiliations:** 1 Department of Cardiology and Hematology, Fukushima Medical University, Fukushima, Japan; 2 Department of Advanced Cardiac Therapeutics, Fukushima Medical University, Fukushima, Japan; Kurume University School of Medicine, Japan

## Abstract

**Background:**

Sleep-disordered breathing (SDB) is associated with adverse outcomes in patients with chronic heart failure (CHF). Additionally, heart rate turbulence (HRT) reflects changes in the sinus cycle length of baroreceptor in response to hemodynamic fluctuations after ventricular premature beat. Recent studies have suggested that HRT as a marker of vagal activity has a predictive value of poor prognosis in CHF patients. However, little is known about the relationship between SDB and HRT in CHF patients.

**Methods and Results:**

In this study, 75 patients with CHF were enrolled. We simultaneously performed Holter ECG during a 24-hr period and portable sleep monitoring at nighttime, and determined the respiratory disturbance index (RDI), HRT (turbulence onset (TO) and turbulence slope (TS)) during that 24-hr period. These patients were divided into two groups based on the presence of severe SDB: Group A (RDI≥30, n = 17) and Group B (RDI<30, n = 58). TS was significantly lower in Group A than in Group B across the 24-hr period (nighttime: 3.6±1.1 vs. 6.9±1.3; daytime: 3.7±0.8 vs. 7.0±1.1; all-day: 3.5±0.7 vs. 6.7±0.9% ms/RR, P<0.05, respectively). TO did not differ between the two groups. Furthermore, there was a significant negative correlation between all-day TS and RDI (R = –0.257, P = 0.027). Moreover, in the multiple regression analysis, RDI was an independent factor to determine all-day TS.

**Conclusions:**

In patients with severe SDB, blunted TS was observed across 24 hours. These results suggest that SDB induce impairment of vagal activity across a 24-hour period and may be associated with poor prognosis in CHF patients.

## Introduction

Despite recent advances in its medical management, chronic heart failure (CHF) still leads to high mortality and morbidity. CHF is characterized by an autonomic imbalance with impaired vagal activity and increased sympathetic activity [1]. Furthermore, reduced vagal activity is associated with increased mortality [2], and recent vagal nerve stimulation for CHF reportedly improved cardiac function and prognosis [3], [4]. Hence, assessment of vagal function is thought to be important in CHF.

About 50% of CHF patients have sleep-disordered breathing (SDB), which consists of obstructive sleep apnea (OSA) and Cheyne-Stokes respiration with central sleep apnea (CSR-CSA). SDB, especially severe SDB, is associated with cardiovascular mortality [5]–[7]. Some studies have demonstrated that SDB is associated with occurrence of ventricular arrhythmias [8], [9] and an adverse prognosis in CHF patients [10], [11]. However, the mechanism of impact of SDB on CHF patients with respect to vagal function remains unclear. Heart rate turbulence (HRT), which presents baroreceptor responses and is a marker of vagal function, is an independent predictor of mortality in CHF patients [12]–[15]. Therefore, we sought to clarify the relationship between the severity of SDB and vagal function (HRT) in CHF patients.

## Methods

### Study subjects and study protocol

This study enrolled 112 consecutive patients with CHF who were referred for an overnight test with a portable sleep monitor and a 24-hr Holter ECG test, regardless of SDB symptoms, at Fukushima Medical University. Inclusion criteria were (1) the presence of symptomatic CHF in New York Heart Association class II–III [16], (2) the enforcement of standard pharmacotherapy (including β-blockers), and (3) stable clinical status, which was defined as receiving optimal medical therapy and being without worsening of heart failure for at least 2 months prior to study enrollment. Exclusion criteria were: (1) the presence of atrial fibrillation or a pacemaker implantation, (2) a few ventricular premature beats (less than 10 beats), (3) acute coronary syndrome, and (4) recent SDB treatment. In this study, patients with atrial fibrillation (n = 17), pacemaker implantation (n = 9), and a few ventricular premature beats (n = 11) were excluded. Finally, we analyzed 75 patients.

We performed simultaneous overnight portable sleep monitoring and 24-hr Holter ECG monitoring. Standard Holter ECG recorders (LS-300, Fukuda Denshi Co., Ltd., Tokyo, Japan) were used to acquire data. Two independent physicians, each one blinded to the results of the other, analyzed polygraphy and Holter ECG. Written informed consent was obtained from all study subjects. The study protocol was approved by the Ethical Committee of Fukushima Medical University.

### Portable sleep monitor

All subjects underwent overnight polygraphy with the use of standard techniques [17]. Overnight sleep study was performed using a cardiopulmonary monitoring (type 3 polygraph) system (LS-300, Fukuda Denshi Co., Ltd., Tokyo, Japan) which monitored the electrocardiogram, thoracoabdominal motion, and nasal airflow by an airflow pressure transducer, and arterial oxyhemoglobin saturation (SpO_2_) by pulse oximetry as previously reported [17]. Apnea was defined as an absence of airflow for more than 10 sec. Hypopnea was defined as a >30% reduction in monitored airflow accompanied by a decrease in SpO_2_>3% [18]. Standard definitions for OSA and CSA were made based the presence or absence of rib cage and abdominal excursions with an absence of airflow. The respiratory disturbance index (RDI) was defined as the number of apneas and hypopneas per hour during the time in bed. All subjects were divided into two groups based on the presence or absence of severe SDB [5]–[7] by a portable sleep monitor: Group A (RDI≥30/h, n = 17) and Group B (RDI<30/h, n = 58). The major polygraphic parameters investigated were RDI, central-RDI, obstructive-RDI, lowest pulse oxygen saturation (lowest SpO_2_), and mean pulse oxygen saturation (mean SpO_2_) [17]. These data were visually inspected and scored by a single experienced laboratory technician who was blinded to the other results.

### Measurement of HRT and HRV

All patients underwent 24-hr Holter ECG and we obtained their HRT and heart rate variability (HRV) parameters as previously reported [13], [19]. ECG signals were obtained from a Holter system (LS-300, Fukuda Denshi Co., Ltd., Tokyo, Japan) at a sampling frequency of 125 Hz. The Holter automatic arrhythmia analysis system (SCM8000, Fukuda Denshi Co., Ltd., Tokyo, Japan) identified all the R wave positions and excluded the abnormal beats such as ventricular ectopic beats, supraventricular ectopic beats, and artifacts. HRT was defined as abnormal according to the definition proposed by Schmidt et al [19]. HRT parameters were analyzed across a 24-hour period and during 2 intervals (in a 24-hour period, during daytime, and in sleep time defined by the behavior records of patients). The turbulence onset (TO) was defined as the difference between the mean of the first 2 sinus RR intervals preceding the ventricular premature complex (VPC) and the mean of the subsequent two sinus RR intervals, expressed as a percentage. TO abnormality was defined as being more than 0% [13], [19], [20]. Turbulence slope (TS) was defined as the maximum positive value of the slope of a regression line assessed over any sequence of five subsequent sinus-rhythm RR intervals within the first 20 sinus-rhythm intervals after VPC. If more than one positive slope occurred in this period, the first positive slope was used. The value of TS was expressed in milliseconds per RR interval. TS abnormality was defined as less than 2.5 ms/RR [13], [19], [20]. When using the two HRT parameters simultaneously, patients were categorized as having both HRT parameters normal (HRT category 0), one HRT parameter abnormal (HRT category 1), or both HRT parameters abnormal (HRT category 2), as previously reported [13], [20]. VPCs were excluded if they failed to satisfy the following criteria: (1) The VPC should occur in isolation from the normal sinus beat occurring at least 12 beats before the VPC, and at least 20 beats after the VPC. (2) The VPC RR maximum and minimum intervals should be greater than 20% and less than 20%, respectively, of the mean of the five preceding normal sinus RR intervals. (3) All RR intervals for the 12 beats before the VPC and 20 beats after the VPC should be greater than 300 ms and less than 2000 ms in duration, respectively. (4) All normal sinus RR intervals 12 beats before the VPC and 20 beats after the VPC should be within 20% of the mean RR interval of all beats in the 24-h ECG recording. (5) Any change in adjacent normal sinus RR intervals, 12 beats before the VPC and 20 beats after the VPC, should not be greater than 200 ms. In our data, the intra- and inter-observer variability (the coefficient of variation) to determine TS were 5.5% and 4.7%, respectively. The intra- and inter-observer variability to determine TO were 1.0% and 0.9%, respectively.

In time domain analysis, standard deviation of all NN intervals (SDNN), standard deviation of the averages of the entire recording (SDANN), the square root of the mean of the sum of the squares of differences between adjacent NN intervals (RMSSD), the number of pairs of adjacent NN intervals differing by more than 50 ms in the entire recording (NN50), and the NN50 count divided by the total number of all NN intervals (pNN50) were measured across a 24-hour period [21]. In power spectral analysis, the variance of NN intervals over the temporal segment (TP), power in the very low frequency component (VLF; <0.04 Hz), low-frequency component (LF; 0.04–0.15 Hz), high-frequency component (HF; 0.15–0.40 Hz), and LF to HF ratio (LF/HF) were measured across a 24-hour period [21]. Spectral powers were expressed in ms^2^ [21].

### Measurement of laboratory and echocardiographic data

Blood samples were obtained the morning after the polygraphy, while the patient was in the supine position in the fasting state. Plasma BNP level was measured using a specific immunoradiometric assay (Shionoria BNP kit, Shionogi, Osaka, Japan). Estimated glomerular filtration rate (eGFR) was measured by the Modification of Diet in Renal Disease formula [22]. Echocardiography was performed using the standard techniques by an experienced echocardiographer at the echo laboratory in our hospital during daytime. Echocardiographic parameters investigated included left ventricular ejection fraction (LVEF) and right ventricular fractional area change (RV-FAC) [23]. The LVEF was calculated using a modification of the Simpson’s method. The RV-FAC, defined as (end diastolic area-end systolic area)/end diastolic area×100, is a measure of right ventricular systolic function [23]. All recordings were performed on ultrasound systems (ACUSON Sequoia, Siemens Medical Solutions USA, Inc., Mountain View, CA, USA). In our data, the intra- and inter-observer variability to determine LVEF were 4.3% and 2.5%, respectively.

### Statistical analysis

Normally distributed data are presented as mean ± SD, and non-normally distributed data are presented as median (inter-quartile range). Categorical variables are expressed as frequencies and relative frequencies. HRT and HRV data are presented as mean ± SE. Characteristics between the two groups were compared using the independent Student’s *t*-test for normally distributed data and the Mann-Whitney U test for non-normally distributed data for continuous variables, and the chi-square test was used for categorical variables. Correlations between RDI and TS were assessed using Spearman correlation analysis. Multivariable regression analysis was used to determine factors related to all-day TS. To prepare for potential confounding, we introduced the following factors, known to affect the TS into models: age, gender, NYHA functional class, heart rate (all-day), ischemic etiology, β-blockers therapy, eGFR, LVEF, and RDI. Parameters with statistical significance in the univariable analysis (P<0.10) were included in the multivariable analysis. A value of P<0.05 was considered significant for all comparisons. Finally, A value of P<0.05 was considered significant. These analyses were performed using a statistical software package (SPSS ver. 21.0, IBM, Armonk, NY, USA).

## Results

### Clinical characteristics of study subjects

There were 17 patients with severe SDB (Group A RDI≥30/h) and 58 patients with none to moderate SDB (Group B; 0≤RDI<30/h). Comparisons of clinical characteristics between Group A and Group B are shown in Table 1. Standard pharmacotherapy including angiotensin-converting enzyme inhibitors, angiotensin II receptor blockers, and β blockers were given to a majority of patients. There were no significant differences in age, gender, NYHA class, medications taken, laboratory data, and echocardiographic data between the two groups. RDI, central-RDI, and obstructive-RDI were significantly higher, and lowest SPO_2_ and mean SPO_2_ were significantly lower in Group A than in Group B.

**Table 1:** Clinical characteristics, HRT, and HRV parameters (N = 75).

	Group A	Group B	P-value
	Severe SDB (N = 17)	Non to moderate SDB (N = 58)	
Age (years)	64.0±10.7	57.3±13.6	0.096
Male (n, %)	13 (76.5)	45 (77.6)	0.579
Body mass index	25.1±6.2	24.2±4.1	0.497
NYHA functional class (I/IIs/IIm/III/IV)	0/1/7/9/0	0/7/24/27/0	0.747
Systolic blood pressure (mmHg)	122.2±18.6	119.0±16.8	0.612
Diastolic blood pressure (mmHg)	75.4±10.7	71.9±12.9	0.440
Heart rate (bpm)	71.7±20.5	66.4±12.6	0.328
Ischemic etiology	9 (52.9)	37 (63.8)	0.297
Medication			
ACE inhibitors/ARB (n, %)	12 (70.6)	44 (75.9)	0.440
β-blockers (n, %)	16 (94.1)	53 (91.4)	0.588
Diuretics (n, %)	12 (70.6)	31 (53.4)	0.164
Data			
Hemoglobin (g/dl)	14.1±1.5	13.5±1.9	0.191
PO_2_ (mmHg)	97.7±12.6	92.4±32.5	0.599
PCO_2_ (mmHg)	38.0±4.2	40.3±9.5	0.444
BNP (pg/ml)†	69.9 (102)	185.1 (381)	0.128
eGFR (ml/min/1.73 cm^2^)	70.5±32.5	67.8±31.5	0.792
C-reactive protein (mg/dl)†	0.16 (1)	0.06 (0)	0.144
Triglyceride (mg/dl)	123.8±32.1	114.8±38.2	0.215
Total cholesterol (mg/dl)	183.6±28.9	178.9±51.6	0.805
Low-density cholesterol (mg/dl)	116.2±34.5	107.7±39.3	0.514
High-density cholesterol (mg/dl)	45.5±9.1	47.1±14.7	0.716
Fasting blood glucose (mg/dl)	113.4±33.6	112.2±35.3	0.453
HbA1c (%)	5.7±0.7	5.7±1.5	0.990
LVEF (%)	54.7±15.1	49.2±14.5	0.204
RV-FAC (%)	38.5±7.2	38.3±14.9	0.984
Epworth sleepiness scale	6.2±3.5	5.4±3.6	0.408
Polygraph			
RDI (/h)	44.1±16.9	15.6±8.3	<0.001
Central-RDI (/h)†	18.4 (15.4)	1.5 (2.6)	<0.001
Obstructive-RDI (/h)†	21.8 (19.6)	6.4 (10.5)	0.026
Lowest SPO_2_ (%)	73.2±12.6	83.9±5.9	<0.001
Mean SPO_2_ (%)	94.8±3.1	96.3±2.8	0.046
SDB severity (none/mild/moderate/severe)	0/0/0/17	5/24/29/0	<0.001
HRT parameters			
Nighttime TS (ms/RR)	3.6±1.1	6.9±1.3	0.035
Daytime TS (ms/RR)	3.7±0.8	7.0±1.1	0.022
All-day TS (ms/RR)	3.5±0.7	6.7±0.9	0.010
TS all-day abnormality (<2.5 ms/RR)	9 (52.9)	13 (22.4)	0.019
Nighttime TO (%)	–0.0060±0.0034	–0.0044±0.0021	0.693
Daytime TO (%)	–0.0088±0.0069	–0.0081±0.0033	0.923
All day TO (%)	0.0000±0.0021	–0.0056±0.0025	0.240
HRT category (0/1/2)	7/9/1	39/19/0	0.043
HRV time domain			
SDNN (ms)	64.6±7.3	108.2±4.9	<0.001
SDANN (ms)	41.9±5.2	89.5±5.0	<0.001
RMSSD (ms)	20.9±2.9	24.9±1.5	0.200
pNN50 (%)	3.8±1.2	4.9±0.9	0.528
HRV frequency domain			
TP (ms^2^)	2369.8±513.7	4105.8±393.8	0.011
VLF (ms^2^)	1213.5±265.7	1697.2±156.5	0.127
LF (ms^2^)	223.0±72.8	333.2±37.0	0.189
HF (ms^2^)	141.2±41.8	191.1±27.0	0.324
LF/HF	1.63±0.29	2.15±0.19	0.149

HRT, heart rate turbulence; HRV, heart rate variability, NYHA, New York Heart Association; ACE, angiotensin-converting enzyme; ARB, angiotensin II receptor blocker; BNP, B-type natriuretic pepide; eGFR, estimated glomerular filtration; LVEF, left ventricular ejection fraction; RV-FAC, right ventricular fractional area change; RDI, respiratory disturbance index; Lowest SpO_2_, lowest oxyhemoglobin saturation; Mean SpO_2_, mean oxyhemoglobin saturation; TS, turbulence slope; TO, turbulence onset; SDNN, standard deviation of all NN intervals; SDANN, standard deviation of the averages of the entire recording; RMSSD, the square root of the mean of the sum of the squares of differences between adjacent NN intervals; pNN50, number of pairs of adjacent NN intervals differing by more than 50 ms in the entire recording count divided by the total number of all NN intervals; TP, variance of NN intervals over the temporal segment; VLF, power in the very low frequency component; LF, power in the low-frequency component; HF, power in the high-frequency component.

† Data are presented as median (inter quartile range).

### HRT and HRV parameters in CHF with severe SDB

**Figure 1:**
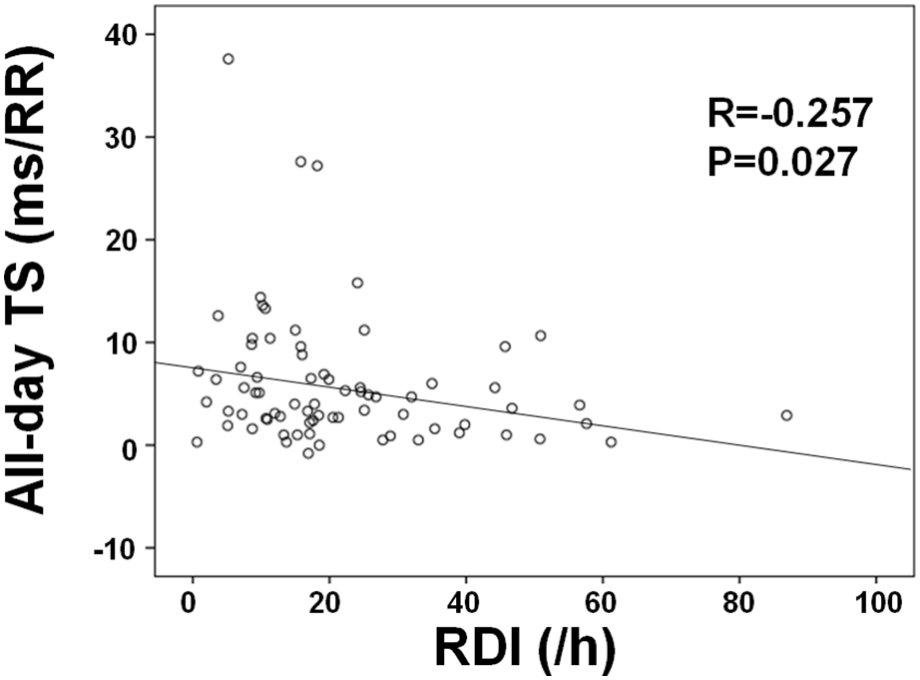
Correlations between all-day TS and RDI in CHF patients.

Table 1 demonstrates comparisons of HRT and HRV parameters between Group A and Group B. TS was significantly lower in Group A than in Group B during the 24-hr period (nighttime: 3.6±1.1 vs. 6.9±1.3; daytime: 3.7±0.8 vs. 7.0±1.1; all-day: 3.5±0.7 vs. 6.7±0.9% ms/RR, P<0.05, respectively). TO did not differ between the two groups. There were more patients with TS abnormality (P = 0.019) and HRT category 1 or 2 (P = 0.043) in Group A than in Group B. Furthermore, SDNN (64.6±7.3 vs. 108.2±4.9 ms, P<0.001), SDANN (41.9±5.2 vs. 89.5±5.0 ms, P<0.001), and TP (1213.5±265.7 vs. 1697.2±156.5 ms^2^, P = 0.011) were significantly lower in Group A than in Group B. In contrast, RMSSD, pNN50, VLF, LF, and HF did not differ between the two groups. Furthermore, as shown in Figure 1, there was a significant negative correlation between all-day TS and RDI (R = –0.257, P = 0.027). Moreover, in the multiple regression analysis (Table 2), the independent factors to determine all-day TS were age, heart rate, LVEF and RDI.

**Table 2:** Multiple regression analysis to determine factors related to all-day TS.

Factors	Univariable analysis		Multivariable analysis	
	β coefficient	p value	β coefficient	p value
Age	–0.234	0.043	–0.300	0.016
Male	–0.036	0.759		
NYHA functional class	0.131	0.261		
Heart rate (all-day)	–0.223	0.054	–0.244	0.041
Ischemic etiology	–0.060	0.609		
β-blockers	–0.068	0.564		
Estimated GFR	0.199	0.283		
LVEF	0.259	0.039	0.361	0.004
RDI	–0.240	0.038	–0.251	0.033

These factors are based on a linear regression analysis.

Estimated GFR, estimated glomerular filtration; LVEF, left ventricular ejection fraction; RDI, respiratory disturbance index.

## Discussion

In this study of CHF patients with severe SDB, blunted TS was observed not only at nighttime but also across a 24-hour day. Furthermore, in the multiple regression analysis, RDI was an independent factor to determine all-day TS in CHF patients. This is, to our knowledge, the first study to show that SDB may induce impairment of vagal activity across a 24-hour period in CHF patients.

### Vagal Activity, Heart Rate Turbulence and CHF

Monitoring heart rate behavior and response is especially important for CHF patients because early autonomic nervous system dysfunction and neurohormonal activation play a dominant role in the progression and prognosis of this disease. [1] Impaired vagal activity is associated with increased mortality [2]. HRT is considered to be a vagally mediated phenomenon, non-invasively reflecting baroreflex sensitivity, which is frequently impaired in patients with CHF [24]. HRT is a measurement of the short-term oscillation of sinus cycle following a VPC, and VPCs are considered to lead to a deviation from the pressure set point of the barorecepter reflex for at least one beat [13]. TO measures the degree of early overshoot deceleration that occurs after a single VPC-induced pause, and TS indicates how fast the RR interval changes after the pause. In healthy humans, the unloading of baroreceptors is considered to lead to a transient withdrawal of cardiac vagal efferent traffic and sympathoactivation, which in turn leads to cardio-acceleration. Sympathetically mediated overshoot of blood pressure then leads to deceleration of sinus rate via increased vagal traffic. Such HRT measurements seem to evaluate both parasympathetic and sympathetic autonomic regulatory mechanisms in response to a defined stimulus. There are limited data regarding the association between TS and severity of CHF [12], [13], [20], [25]. Koyama et al. reported that TS was lower in CHF patients who subsequently died or were being admitted to hospital with worsening heart failure [12]. Moore et al. reported that TS is an independent predictor of death due to decompensated heart failure in ambulant heart failure patients [13]. Moreover, Cygankiewicz et al. reported that abnormal TS and HRT category 2 were independently associated with increased all-cause mortality, sudden death, and heart failure death after adjustment for clinical covariates in multivariate analysis [14].

### Impacts of SDB on HRT and HRV in CHF

SDB (especially OSA) is characterized by recurrent hypoxia, arousal, and the generation of exaggerated negative intrathoracic pressure, which increases sympathetic nervous activity, reduces cardiac parasympathetic activity, and causes repetitive surges in heart rate, blood pressure, and left ventricular preload and afterload, resulting in decreasing stroke volume [26]. Abnormal HRT and HRV have been reported in SDB patients without cardiovascular diseases [27]–[29]. Yang et al. reported that nighttime TS correlated inversely with the severity of SDB in SDB patients without cardiovascular disease [28]. No significant correlation of TO and the severity of SDB was observed [28]. Sleep is associated with dominant vagal activity in healthy subjects, and SDB may alter this physiologic event. Aytemir et al. also reported that impairment of TS was observed in SDB patients [29]. TS is also affected by several factors such as age, heart rate, NYHA functional class, β-blockers, and LVEF. These factors may be cause of individual differences of TS [12], [13], [20], [25]. However, in our multiple regression analysis, RDI was an independent factor to determine all-day TS in CHF patients. It has been reported that HRV is an appropriate parameter for estimating autonomic nervous control across a 24-hour period in CHF patients [30], [31], and there is convincing evidence that autonomic nervous imbalance caused by SDB contributes to increased mortality in CHF patients [32]. Our data were concordant with previous reports showing that SDNN and SDANN were also significantly lower in HF patients with SDB [9], [33]. Thus, a blunted HRT and HRV oscillation indicated the impairment of autonomic nervous control and an increased risk for ventricular tachyarrhythmias and sudden cardiac death during the 24-hour period.

In the present study, we first demonstrated impairment of TS in CHF patients with severe SDB, and the severity of SDB is an independent factor of impaired vagal activity during the 24-hour period, regardless of age, heart rate, etiology, cardiac function and β-blocker therapy. These data may provide us with novel mechanistic and therapeutic insights to understand the clinical impacts of SDB on CHF patients. Thus, the impaired vagal activity protective approach by treatment of SDB has clinically important implications for improving the prognosis of patients with CHF.

### Limitations

Our study has some limitations. First, our study was just observational nature and had no control groups such as patients with only SDB without CHF, and patients without SDB and CHF. Second, the patients with atrial fibrillation were excluded, because HRT could not be determined in the presence of atrial fibrillation. Third, the gold standard for diagnostic testing of SDB is full-channel polysomnography, which provides detailed information about complete differentiation of the types of apnea. Therefore, our differentiation of SDB by portable sleep monitor might have been less reliable than by full polysomnography. Finally, the sample size was relatively small. Large-scale clinical trials may be required to confirm the impact of SDB on HRT in CHF patients.

## Conclusions

In patients with severe SDB, blunted TS was observed across a 24-hour period. Furthermore, SDB is an independent factor of impaired TS. Common pathological mechanisms underlying SDB and CHF, leading to baroreceptor reflex suppression, may cause abnormal heart rhythm turbulence in CHF patients with severe SDB. These results suggest that SDB induce impairment of vagal activity across a 24-hour period and may be associated with poor prognosis in CHF patients.
